# Evaluation of Biorefinery Products From *Microchloropsis gaditana*, *Isochrysis galbana*, and *Arthrospira platensis* in Juvenile Gilthead Seabream (*Sparus aurata*): Effect on Growth, Nutrient Utilization, Chemical Composition, Metabolism, and Intestinal Functionality

**DOI:** 10.1155/anu/1957808

**Published:** 2026-08-06

**Authors:** Sara Flores-Moreno, Alba Galafat, Antonio J. Vizcaíno, Alejandro Blázquez-Durán, Verónica de las Heras, Marta Román, Paula Simó-Mirabet, Ismael Hachero-Cruzado, Elvira Navarro-López, Miguel Angel González-Cardoso, Mari Carmen Cerón-García, Juan Antonio Martos-Sitcha, Francisco Javier Alarcón-López

**Affiliations:** ^1^ Department of Biology and Geology, CEI·MAR, University of Almeria, 04120, La Canada de San Urbano, Almeria, Spain, ual.es; ^2^ LifeBioencapsulation S.L., 04131, Almeria, Spain; ^3^ Department of Biology, Faculty of Marine and Environmental Sciences, University of Cadiz, 11519, Puerto Real, Cadiz, Spain, uca.es; ^4^ Central Research Services for Marine Aquaculture (SCI-CM), University of Cadiz, 11519, Puerto Real, Cadiz, Spain, uca.es; ^5^ Andalusian Institute for Research and Training in Agricultural, Fisheries, Food and Organic Production, El Toruño Center, 11500, El Puerto de Santa Maria, Cadiz, Spain; ^6^ “Blue Growth”, IFAPA-ICMAN Associated Unit, 11500, El Puerto de Santa Maria, Cadiz, Spain; ^7^ Department of Chemical Engineering and CIAIMBITAL, University of Almeria, 04120, Almeria, Spain, ual.es

**Keywords:** aquafeed, *Arthrospira platensis*, fatty acids, *Isochrysis galbana*, microchloropsis gaditana, *Sparus aurata*

## Abstract

The present study evaluated the effects of replacing fish oil with microalgal oil on growth performance, nutrient utilization, chemical composition, metabolism and gut functionality in *Sparus aurata*. Three experimental diets were formulated with increasing levels of oils derived from the microalgae *Microchloropsis gaditana* and *Isochrysis galbana* for replacing fish oil: 50% (MO50), 75% (MO75) to 100% (MO100) of total oil. All diets were also supplemented with a protein hydrolysate obtained from *Arthrospira platensis* to partially replace fishmeal, as well as with an algal carotenoid extract. A total of 198 fish (initial body weight 10.5 ± 0.4 g) were randomly distributed into 12,400 L tanks and fed the experimental diets *ad libitum* for 83 days under controlled conditions. Growth performance, feed efficiency and somatic indices were assessed, while muscle proximate composition, fatty acid and carotenoid profiles, plasma metabolites, and tissue glycogen were analyzed using standard biochemical and chromatographic techniques. Digestive enzyme activities (trypsin, chymotrypsin, leucine aminopeptidase, alkaline phosphatase) and well as intestinal histology were also evaluated. The results evidenced that use of microalgal oil did not affect fish growth. Fish fed diets supplemented with microalgal products tended to reduce muscle lipid content. Fish fed diets devoid fish oil showed an increase in eicosapentaenoic acid (EPA) content when microalgal oil was included. Plasma cortisol did not vary among experimental groups. The activity of foregut trypsin and alkaline phosphatase increased with the use of microalgal oil, while in the hindgut trypsin and leucine aminopeptidase activities were enhanced. The use of algal products promoted longer intestinal villi and reduced *lamina propria* thickness in the distal intestine. Overall, the results indicate that total replacement of fish oil with microalgal oil has no detrimental effects on growth performance, and exerts positive effect on specific fatty acids and intestinal functionality.

## 1. Introduction

The long‐term sustainability of the aquaculture sector is closely linked to the identification and development of viable alternatives to fishmeal and fish oil, which have traditionally been used as the primary sources of protein and lipids in aquafeed formulations [1, 2]. Despite significant advances in aquaculture nutrition, commercial feeds still rely heavily on these conventional inputs [2–4], raising serious environmental sustainability concerns due to the pressure exerted on marine ecosystems and the exploitation of finite oceanic resources. Moreover, stagnation in global fishmeal and fish oil production, contrasted with the steadily increasing demand from the aquaculture, pharmaceutical, and nutraceutical industries, has resulted in increasingly limited availability and heightened price volatility [5–7].

In this context, microalgae have emerged as promising candidates for the partial or complete replacement of fishmeal and fish oil in aquafeeds, thereby contributing to the reduction of the structural dependency between aquaculture and capture fisheries [7–10]. From a nutritional perspective, microalgae are characterized by a high protein content [11, 12] and a balanced and complete amino acid profile, including all essential amino acids required by fish. This ensures a comprehensive nutritional contribution capable of meeting the physiological demands of farmed species [13, 14]. Such amino acid profiles are comparable to those of many conventional protein sources used in aquaculture, positioning microalgae as a promising and functional alternative to fishmeal [10, 15, 16].

In addition to their high protein content, microalgae are also characterized by their richness in high‐quality lipids, making them a sustainable and efficient source for replacing fish oil in aquafeeds [2, 15]. These lipids are particularly rich in long‐chain omega‐3 polyunsaturated fatty acids (LC‐PUFAs), such as eicosapentaenoic acid (EPA) and docosahexaenoic acid (DHA) [17, 18], which are essential for optimal growth and development, immune function, and overall welfare in fish [2, 19, 20].

Moreover, microalgae represent an important source of bioactive compounds with functional properties relevant for aquatic organisms [12, 15, 21, 22]. Among these compounds, carotenoids have received increasing attention in aquaculture nutrition, not only for their role as natural pigments responsible for muscle coloration, a commercially desirable trait, but also for their potent antioxidant, anti‐inflammatory, and immunomodulatory properties [23–25]. The inclusion of microalgal carotenoids may enhance the endogenous antioxidant capacity of fish, strengthen epithelial barriers, and positively modulate innate immune responses, which are key aspects for addressing the challenges associated with intensive aquaculture systems [26–28]. Among the most promising microalgal species are *Microchloropsis gaditana*, *Isochrysis galbana*, and *Arthrospira platensis*, all of which exhibit favorable nutritional profiles for aquaculture diet formulation.

*M. gaditana* is particularly recognized for its exceptional lipid accumulation capacity, reaching up to 48% of dry biomass, with a high concentration of long‐chain polyunsaturated fatty acids, especially EPA, as well as antioxidant carotenoids such as violaxanthin and vaucheriaxanthin [2, 29, 30]. These attributes position *M. gaditana* as an efficient and sustainable source of lipids and essential fatty acids for replacing fish oil in aquafeed formulations [2, 31, 32].

*I. galbana* is notable for its high DHA content, making it a valuable resource for enhancing the lipid quality of aquafeeds and promoting fish health and welfare [33–35]. In addition, *I. galbana* is a rich source of carotenoids and other bioactive compounds with functional properties relevant to aquaculture nutrition [36]. Among its carotenoid content, fucoxanthin is particularly noteworthy due to its antioxidant activity, along with polyphenols and glycolipids, which exhibit anti‐inflammatory properties and may modulate immune responses in aquatic organisms [24, 34, 37]. Moreover, extracts from *I. galbana* have demonstrated antimicrobial activity against pathogenic bacteria and fungi, suggesting their potential to enhance fish health and disease resistance [24, 28].

On the other hand, *Arthrospira platensis* is highly valued in aquaculture for its exceptional nutritional profile. This filamentous cyanobacterium is characterized by a high protein content, which can range from 50% to 70% of dry weight, along with a balanced amino acid profile capable of meeting the nutritional requirements of fish [12, 38]. Beyond its protein contribution, *A. platensis* is a significant source of functional compounds, including bioactive peptides, essential fatty acids, water‐soluble vitamins, and natural pigments. These compounds have been associated with immunostimulatory, antioxidant, and metabolism‐enhancing effects in farmed fish [39, 40]. These properties can be further enhanced through enzymatic protein hydrolysis pretreatment of microalgal biomass, a strategy that significantly promotes the release of peptides and free amino acids [40–42]. These low‐molecular‐weight peptides exhibit greater solubility and bioavailability and can be more efficiently absorbed by enterocytes, which is associated with improved digestibility and palatability of aquafeeds [41–43].

In this context, the integration of high‐value products obtained from a microalgal biorefinerey into aquafeeds represents an innovative and promising nutritional strategy to promote more sustainable, resilient, and physiologically and environmentally efficient aquaculture production systems. In this study, we hypothesize that the complete replacement of fish oil with algal oil is feasible when the diet is supplemented with an algal protein hydrolysate partially substituting fishmeal, together with an algal carotenoid extract. Therefore, the main objective of this study was to evaluate the effects of partially replacing fishmeal with a microalgal protein hydrolysate, fully substituting fish oil with oil from a microalgal blend, and including a microalgal carotenoid‐rich extract on growth performance and feed utilization, proximate composition, fatty acid and carotenoid profiles of fish muscle, intermediary metabolism, and digestive functionality in juvenile gilthead seabream (*Sparus aurata*).

## 2. Materials and Methods

### 2.1. Microalgae Oils

*M. gaditana* and *I. galbana* strains were obtained from the Marine Culture Collection of the Institute of Marine Sciences of Andalucía (CSIC), Cádiz, Spain. These strains were cultured at the pilot microalgae facility of the University of Almería (Spain) under continuous conditions, with a dilution rate of 0.2 day^−1^, in flat panel and tubular photobioreactors [44]. Once the cultures reached a steady state, they were harvested. Biomass was recovered by centrifugation at 9000×*g* for 5 min, washed with a 0.5 M aqueous ammonium bicarbonate solution, and freeze‐dried using a Cryodos 50 instrument (Telstar, Spain) prior to lipid extraction. The fatty acid extract (saponifiable lipid fraction) was separated from the carotenoid extract (unsaponifiable lipid fraction), as detailed in Sales et al. [29]. The fatty acid profiles of the *M. gaditana* and *I. galbana* oils and the blend oil are detailed in Table 1. The carotenoid profile of the unsaponifiable fraction is shown in Table 2. A protein hydrolysate (70% crude protein) obtained from *Arthrospira platensis* biomass according to a modification of Soriano‐Jerez et al. [44] was also used in the elaboration of experimental diets. The concept for obtaining different high‐value products derived from a microalgal biorefinery was proposed recently by González‐Cardoso et al. [45].

**Table 1:** Fatty acid profile (% total fatty acids) of the microalgal oil used.

Fatty acid (%)	*M. gaditana* oil	*I. galbana* oil	Blend of microalgal oils (40:60)
14:0	5.9	14.3	4.1
16:0	24.3	13.5	27.4
16:1n7	24.5	4.6	6.9
18:0	0.6	1.1	0.8
18:1n9	5.6	10.9	1.8
18:2n6	3.2	6.2	5.0
18:3n3		9.8	5.9
18:4n3		22.5	13.5
20:1n9		1.5	0.9
20:4n6, ARA	4.3		1.7
20:5n3, EPA	23.2	1	9.9
22:5n3		2.1	1.3
22:6n3, DHA		10.8	6.5
Others	8.4	1.7	4.4

**Table 2:** Carotenoid profile (%) in the unsaponifiable fraction of the microalgae blend (*M. gaditana* and *I. galbana*).

Carotenoid	%
Neoxanthin	8.5
Violaxanthin/Fucoxanthin	48.1
Anteroxanthin	9.6
Vaucheroxanthin	3.8
Zeaxanthin	0.9
Diadinoxanthin	6.7
Vaucheroxanthin ester	1.5
*β*‐carotene	21.0

### 2.2. Experimental Diets

Three isonitrogenous (45% dry matter [DM]) and isolipidic (18% DM) aquafeeds were prepared at the CEI·MAR‐University of Almeria facilities (Servicio de Piensos Experimentales, Almería, Spain). The experimental diets were formulated with a blend of *M. gaditana* and *I. galbana* oils at different inclusion levels; 50% (MO50), 75% (MO75), and 100% (MO100) of total oils. Each diet was supplemented with 0.5% of the nonsaponifiable fraction, rich in carotenoids and derived from the extraction of microalgal oils (Table 2), and 5% of fishmeal was replaced with a microalgal protein hydrolysate. Briefly, all dietary ingredients were combined in an industrial mixer and processed using a UPZ 100 hammer mill (Hosokawa‐Alpine, Augsburg, Germany) to achieve a particle size of 0.25 mm. The resulting ingredient blend was then processed by extrusion using an Evolum 25 twin‐screw extruder (Clextral, France) to produce 2.5 mm diameter pellets. The extruder configuration was set with a temperature profile of 94°C 94°C, 94°C, 95°C, and 90°C, respectively. The extruded pellets were dried at 29°C in a 12 m^3^ chamber (Airfrio, Almeria), cooled to room temperature, and subsequently coated with vegetable oil using a Pegasus PG‐10VC vacuum coater (Dinnissen, the Netherlands). Finally, the aquafeeds were packed in airtight bags and stored at −20°C until use. The ingredients, proximal composition, fatty acid, and carotenoids profiles of the experimental diets are detailed in Tables 3 and 4, respectively.

**Table 3:** Ingredients and proximate composition (g 100 g^−1^ dry matter [DM]) of the experimental diets.

Ingredients	MO50	MO75	MO100
Fishmeal LT94^a^	15.0	15.0	15.0
Microalgal protein hydrolysate^b^	5.0	5.0	5.0
Squid meal^c^	2.0	2.0	2.0
CPSP90^d^	1.0	1.0	1.0
Krill meal^e^	2.0	2.0	2.0
Wheat gluten^f^	10.0	10.0	10.0
Soybean protein concentrate^g^	10.0	10.0	10.0
Soybean meal^h^	10.0	10.0	10.0
Pea protein concentrate^i^	8.0	8.0	8.0
Fish oil^j^	5.0	2.5	
Blend of microalgal oil^k^	5.0	7.5	10.0
Vegetable oil^l^	4.5	4.5	4.5
Soybean lecithin^m^	1.0	1.0	1.0
Wheat meal^n^	16.7	16.7	16.7
Monoammonium phosphate^o^	0.5	0.5	0.5
Lysine^p^	1.2	1.2	1.2
Methionine^q^	0.5	0.5	0.5
Vitamin and mineral premix^r^	2.0	2.0	2.0
Vitamin C^s^	0.1	0.1	0.1
Carotenoid microalgal extract^t^	0.5	0.5	0.5
Proximal composition (% DM)			
Crude protein	45.1 ± 0.1	45.6 ± 0.3	45.2 ± 0.1
Crude lipid	18.5 ± 0.3	18.8 ± 0.3	18.5 ± 0.4
Ash	6.7 ± 0.1	6.8 ± 0.1	6.8 ± 0.1
Moisture	6.8 ± 0.37	7.2 ± 0.3	7.4 ± 0.5

*Note:* MO50: diet with 50% microalgal oil; MO75: diet with 75% microalgal oil; MO100: diet with 100% microalgal oil.

^a^69.4% crude protein, 12.3% crude lipid (Norsildemel, Bergen, Norway).

^b^Hydrolysate of microalgal proteins, obtained from *Arthrospira platensis* (Department of Chemical Engineering, University of Almeria, Spain).

^c,d,e^Purchased from Bacarel (UK). CPSP90 is enzymatically predigested fishmeal.

^f^78% crude protein (Lorca Nutrición Animal SA, Murcia, Spain).

^g^Soycomil, 60% crude protein, 1.5% crude lipid (ADM, Poland).

^h^50% crude protein, 8% crude lipid (Lorca Nutrition Animal SA, Murcial, Spain).

^i^Pea protein concentrate, 85% crude protein, 1.5% crude lipid (Emilio Peña SA, Spain). Soybean protein concentrate, 50% crude protein, 8% crude lipid (Lorca Nutrition Animal SA, Murcia, Spain).

^j^AF117DHA (Afamsa, Spain).

^k^Microalgal oil from *M. gaditana* and *I. galbana* (Department of Chemical Engineering, University of Almeria, Spain. Method proposed by Sales et al. [22]).

^l^Soybean and rapessed oils (1:2) (Roviroli, Spain).

^m^P700IP (Lecico, DE).

^n^Local provider (Almería, Spain).

^o,p,q^Lorca Nutrición Animal SA (Murcia, Spain).

^r^ *Lifebioencapsulation* SL (Almería, Spain). Vitamins (mg kg^−1^): vitamin A (retinyl acetate), 2,000,000 UI; vitamin D3 (DL‐cholecalciferol), 200,000 UI; vitamin E (Lutavit E50), 10,000 mg; vitamin K3 (menadione sodium bisulfite), 2500 mg; vitamin B1(thiamine hydrochloride), 3000 mg; vitamin B2 (riboflavin), 3000 mg; calcium pantothenate, 10,000 mg; nicotinic acid, 20,000 mg; vitamin B6 (pyridoxine hydrochloride), 2000 mg; vitamin B9 (folic acid), 1500 mg; vitamin B12 (cyanocobalamin), 10 mg vitamin H (biotin), 300 mg; inositol, 50,000 mg; betaine (Betafin S1), 50,000 mg. Minerals (mg kg^−1^): Co (cobalt carbonate), 65 mg; Cu (cupric sulfate), 900 mg; Fe (iron sulfate), 600 mg; I (potassium iodide), 50 mg; Mn (manganese oxide), 960 mg; Se (sodium selenite), 1 mg; Zn (zinc sulfate) 750 mg; Ca (calcium carbonate), 18.6%; (186,000 mg); KCl, 2.41%; (24,100 mg); NaCl, 4.0% (40,000 mg).

^s^TECNOVIT, Spain.

^t^Carotenoid extract obtained from *M. gaditana and I. galbana* (Department of Chemical Engineering, University of Almeria, Spain. Method proposed by Sales et al. [22]).

**Table 4:** Fatty acid (% total fatty acids) profile and carotenoid (mg kg^−1^) content in the experimental diets.

Fatty acid	MO50	MO75	MO100
14:0	2.8	2.8	3.0
16:0	18.9	19.2	20.9
16:1n7	4.2	4.5	5.1
16:2n4	0.4	0.3	0.3
16:3n4	0.3	0.3	0.3
18:0	3.4	2.9	2.3
18:1n7	2.1	2.7	2.4
18:1n9	24.4	21.6	20.5
18:2n6	16.5	16.2	16.7
18:3n3	2.8	2.7	2.8
18:4n3	0.3	0.3	0.4
20:4n6, ARA	1.2	1.3	1.5
20:4n3	0.5	0.4	0.4
20:5n3, EPA	8.8	9.7	11.1
20:1n9	1.1	1.3	0.7
22:5n3	1.0	0.8	0.8
22:6n3, DHA	13.5	13.3	13.8
Neoxanthin	0.3	0.2	0.1
Violaxanthin/fucoxanthin	2.4	2.4	2.5
Lutein			
Anteroxanthin	0.3	0.3	0.2
Vaucheroxanthin	0.2	0.2	0.1
Zeaxanthin	0.9	1.0	1.3
Vaucheroxanthin ester	9.1	9.5	8.6
Cantaxanthin	0.1	0.1	0.1
*β*‐carotene	1.4	1.5	1.6

*Note:* MO50: diet with 50% microalgal oil; MO75: diet with 75% microalgal oil; MO100: diet with 100% microalgal oil.

Abbreviations: ARA, arachidonic acid; DHA, docosahexaenoic acid; EPA, eicosapentaenoic acid.

### 2.3. Animals, Experimental Design, and Fish Sampling

Juvenile gilthead seabream (*Sparus aurata*) were obtained from a commercial hatchery (Cultivos Piscícolas de Barbate S.L., Cádiz, Spain). A total of 198 individuals (mean initial body weight: 10.5 ± 0.4 g) were randomly distributed into nine 400 L tanks, each adjusted to a working volume of 200 L, resulting in an initial stocking density of ~1.20 kg fish m^−3^ (22 fish per tank). Tanks operated under an open flow‐through system and were maintained under controlled conditions: seawater salinity of 38‰, a natural photoperiod corresponding to the February–May period at the experimental facility latitude (36°31′44″ N; ranging from 10 L:14D to 13 L:11D during the feeding period), outlet water oxygen levels above 85% saturation, and a constant water temperature of 18°C–19°C. Fish were acclimatized for 1 week prior to the start of the feeding trial, during which they were fed a diet free of microalgal oils and carotenoids and *A. platensis* hydrolysate. All experimental procedures were conducted at the indoor wet laboratories of the Central Services for Marine Culture Research (SCI‐CM, CASEM, University of Cadiz, Cadiz, Spain; REGA Operational Code ES11028000312), in strict compliance with the guidelines for experimental procedures in animal research of the Ethics and Animal Welfare Committee of the University of Cadiz, according to Spanish (RD53/2013) and European Union (2010/63/EU) legislation. The Ethics Committee from the Regional Government approved the experiments (Junta de Andalucía, reference number 23/10/2019/176). During the 83‐day feeding period, fish were fed *ad libitum* four times per day, 6 days per week, and feed intake (FI) was recorded weekly for each replicate tank to calculate growth performance parameters. At the end of the trial, all fish were anesthetized with a sublethal dose of 2‐phenoxyethanol (Sigma–Aldrich, 0.5 mL L^−1^ in seawater) and immediately sampled for the final biometric evaluation (body weight and total length). In addition, six fish per tank were randomly selected following a 24 h fasting period, euthanized with a lethal dose of 2‐phenoxyethanol (1 mL L^−1^ in seawater), and immediately sampled. Blood was collected via caudal puncture using heparinized syringes, and plasma was obtained by centrifugation at 3000 × *g* for 20 min at 4°C and stored at −80°C until further analysis. For digestive enzyme activity assessment, intestinal samples were randomly pooled to obtain three enzyme extracts from each experimental tank (*n* = 3 per dietary group). Additionally, intestinal sections from three fish per tank were collected for histological examination by light microscopy.

### 2.4. Growth Performance and Somatic Parameters

The following zootechnical parameters were determined to evaluate the productive performance of the fish: (i) specific growth rate (SGR, % day^−1^) = [100 × (ln final body weight – ln initial body weight)/days]; (ii) weight gain (WG, %) = [100 (final weight – initial weight)/initial body weight]; (iii) FI (g/fish) = aquafeed consumed per fish/days; (iv) feed efficiency (FE, %) = [100 × (weight gain/total FI)]; and (v) condition factor = (100 × body weight (g))/fork length (cm^3^). Additionally, somatic indices were calculated as indicators of the relative allocation of resources to key organs: (i) hepatosomatic index (HSI, %) = (100 × liver weight (g))/total body weight (g); (ii) intestine length index (ILI, %) = (100 × total intestine length (cm))/total fish length (cm); and (iii) mesenteric fat index (MSI, %) = (100 × mesenteric fat weight (g))/total body weight (g).

### 2.5. Chemical Composition and Fatty Acids Analysis

Proximate composition of experimental diets and muscle samples was determined following AOAC [46] standard methods for DM and ash. Crude protein (N × 6.25) was determined by elemental analysis (C: H: N) with a Fisons EA 1108 analyzer (Fisons Instruments, USA). Values were expressed as percentage of dry matter (% DM). Total lipids were determined following the methodology described by Folch et al. [47], using a mixture of chloroform:methanol (2:1 v/v) as solvent. Fatty acid profile was determined by gas chromatography on an HP Series II 4890 instrument (Hewlett Packard, Avondale, PA), following Rodríguez‐Ruiz et al. [48]. Fatty acid methyl esters were prepared using a modified direct transesterification protocol [49] that eliminates the need for prior lipid fraction separation. Carotenoids quantification was performed using a Shimadzu SPDM10AV high‐performance liquid chromatograph (HPLC) (Shimadzu, USA) equipped with a photodiode array detector, according to Cerón‐García et al. [50]. Separation was achieved on a LiChrospher 100 RP‐18 column (5 μm; 4.6 × 150 mm). Sample injection volume was 20 μL. Gradient elution utilized water:methanol (1:4, v/v) and acetone:methanol (1:1, v/v) at 1 mL min^−1^, with absorbance recorded from 360 to 700 nm.

### 2.6. Plasma, Liver, and Muscle Biochemical Analysis

Plasma cortisol levels were determined according to the protocol provided by the commercial Cortisol Enzyme Immunoassay Kit (ArborAssays, NCAL International Standard Kit, DETECTX, K003). Glucose, lactate, triglycerides, and cholesterol in plasma and glucose, lactate, and triglycerides in liver and muscle were measured using commercial kits from SPINREACT (St. Esteve de Bas, Girona, Spain) adapted to 96‐well microplates. The tissue glycogen concentration was quantified following a method based on the technique described by Keppler and Deckler [51]. In this method, glucose released after glycogen breakdown with amyloglucosidase (SIGMA‐ALDRICH A7420) was measured using the same commercial SPINREACT kit by means of glucose determinations. Plasma total protein concentration was determined with a BCA Protein Assay Kit (PIERCE, Thermo Fisher Scientific, USA, #23225) using BSA as the standard. All assays were performed using a POWERWAVE 340 microplate spectrophotometer (Bio‐Tek Instruments, Winooski, VT, USA) and the Gen5 data analysis software for MICROSOFT. Frozen liver and muscle were prepared prior to the analysis of their metabolites. This involved a homogenization process starting with deproteinization using a 0.024 N perchloric acid solution from Merck KGaA. The samples were then homogenized with a highly efficient device, the T 25 digital Ultra‐Turrax. Subsequently, they were neutralized with a basic solution of 1 M potassium bicarbonate (Merck KGaA), resulting in a 15‐fold dilution of tissue weight. The homogenates were subjected to centrifugation at 3250×*g* and 4°C for 30 min. Aliquots of the resulting aqueous phase, which contained the metabolites of interest, were then taken.

### 2.7. Digestive Enzyme Activities

For the analysis of digestive enzyme activities, the intestinal samples were homogenized in distilled water at 4°C (0.5 g mL^−1^). Supernatants were obtained after centrifugation (12,000 × *g*, 12 min, 4°C) and stored at −20°C until further use. Trypsin and chymotrypsin activities were evaluated using 0.5 mM BAPNA and 0.2 mM SAPNA substrates, respectively, in 50 mM Tris‐HCl buffer containing 10 mM CaCl_2_, pH 8.5, following the procedures described by Erlanger et al. [52] and DelMar et al. [53], respectively. Leucine aminopeptidase activity was determined using 2 mM L‐leucine‐p‐nitroanilide (LpNa) in 100 mM Tris‐HCl buffer pH 8.8, according to the method of Pfleiderer [54]. Alkaline phosphatase activity was quantified using p‐nitrophenyl phosphate, as a substrate, and buffer pH 9.5 [55]. A unit of activity (UA) for trypsin, chymotrypsin, and leucine aminopeptidase enzymes was defined as the amount of enzyme that released 1 μmol of p‐nitroanilide (pNA) per min using an extinction coefficient of 8800 M cm^−1^ at 405 nm. For alkaline phosphatase, an UA was set as the amount of enzyme that released 1 μg of nitrophenyl per min using an extinction coefficient of 17.800 M cm^−1^ at 405 nm. All assays were performed in triplicate, and the specific enzyme activity was expressed as units of activity per gram of tissue (UA g^−1^ tissue).

### 2.8. Histological and Morphometric Analysis of the Intestinal Mucosa

Intestinal samples were processed according to standard histological procedures, as described by Vizcaíno et al. [56]. Briefly, samples of tissue were fixed for 24 h in 4% formalin solution at pH 7.2, followed by dehydration and paraffin. Transverse sections of 5 μm thickness were obtained and stained with hematoxylin and eosin (H&E). The sections were examined using an optical microscope (Olympus ix51, Olympus, Barcelona, Spain) equipped with a digital camera (CC12, Olympus Soft Imaging Solutions GmbH, Muenster, Germany). The resulting images were analyzed using specific software called Image J (U.S. National Institutes of Health). From the intestinal sections, the following morphometric parameters were measured: *villi* width (VW), *villi* height (VH), and *lamina propria* thickness (LT). For each slide, 20 measurements were taken, and the procedure was repeated on two slides per fish.

### 2.9. Statistics

The results are expressed in terms of mean and standard error of the mean (SEM). The normality of the distribution and homogeneity of variances of all data were verified and, when necessary, transformations were applied to normalize the data. A one‐way analysis of variance (ANOVA) was performed in which the main factor was the level of dietary supplementation. The significance level used was 5% (*p* < 0.05). In addition, Tukey’s multiple range test was performed to identify significant differences between means. Results of fatty acids were analyzed using orthogonal contrasts to determine the linear effect of increasing dietary level of algal oil. All statistical analyses were performed with GraphPad Prism 8.0 software (Inc., San Diego, CA, USA) for Windows.

## 3. Results

### 3.1. Growth Performance

Table 5 presents the growth performance and zootechnical parameters measured in juvenile gilthead seabream fed the experimental diets. Final body weight did not differ significantly among dietary treatments. In contrast, final body length was significantly greater in fish fed the MO75 and MO100 diets compared with those receiving the MO50 diet. No significant differences were observed among treatments for FI, feed efficiency, weight gain, or specific growth rate. With regard to somatic indices, no significant differences were detected among dietary treatments for condition factor, hepatosomatic index, intestine length index, or mesenteric index.

**Table 5:** Growth performance and zootechnical parameters evaluated in *S. aurata* juveniles fed the different experimental diets.

Parameter	MO50	MO75	MO100	*p*‐Value
Final weight (g)	27.9 ± 5.8	28.3 ± 5.2	28.5 ± 4.2	0.7759
Final length (cm)	11.3 ± 0.1	11.6 ± 0.6	11.6 ± 0.6	0.0671
FI (g/fish)	24.3 ± 0.7	23.8 ± 0.2	24.9 ± 0.3	0.3895
FE (%)	0.7 ± 0.1	0.8 ± 0.1	0.7 ± 0.1	0.6004
WG (%)	164.2 ± 2.7	170.9 ± 2.0	174.4 ± 8.6	0.1869
SGR (%)	1.2 ± 0.1	1.2 ± 0.1	1.2 ± 0.1	0.1861
K (g cm^3^)	1.9 ± 0.1	1.8 ± 0.1	1.8 ± 0.1	0.0623
HSI (%)	1.0 ± 0.0	1.1 ± 0.1	1.1 ± 0.3	0.2431
ILI (%)	105.1 ± 7.7	114.2 ± 6.9	99.4 ± 4.9	0.2990
MSI (%)	0.7 ± 0.1	1.1 ± 0.1	0.9 ± 0.1	0.2040

*Note:* MO50: diet with 50% microalgal oil; MO75: diet with 75% microalgal oil; MO100: diet with 100% microalgal oil. Values are expressed as mean ± SEM (*n* = 3).

Abbreviations: FE, feed efficiency; FI, feed intake; HIS, hepatosomatic index; ILI, intestine length index; K, condition factor; MSI, mesenteric index; SGR, specific growth rate; WG, weight gain.

### 3.2. Proximate Composition of Muscle

The proximate composition of muscle in juvenile gilthead seabream fed the different dietary treatments is presented in Table 6. Total protein content did not differ significantly among dietary treatments, with comparable values observed across all groups. Similarly, no significant differences were detected in muscle lipid content, although numerically lower values were recorded in fish fed the MO100 diet. Ash content also remained unaffected by dietary treatment, showing highly consistent values across groups. Muscle moisture content did not vary among experimental treatments.

**Table 6:** Proximate composition (% dry matter [DM]) of muscle in *S. aurata* juveniles fed the different experimental diets.

Component	MO50	MO75	MO100	*p*‐Value
Total protein	79.0 ± 0.8	78.4 ± 0.6	79.2 ± 0.7	0.5475
Total lipids	15.3 ± 0.6	14.5 ± 1.2	13.2 ± 0.3	0.1557
Ash	5.4 ± 0.1	5.3 ± 0.2	5.3 ± 0.1	0.9475
Moisture	75.3 ± 0.5	75.6 ± 0.7	75.7 ± 0.3	0.6872

*Note:* MO50: diet with 50% microalgal oil; MO75: diet with 75% microalgal oil; MO100: diet with 100% microalgal oil. Values are expressed as mean ± SEM (*n* = 3).

### 3.3. Fatty Acids and Carotenoids Profiles

The muscle fatty acid profile and carotenoid content are detailed in Table 7. An increase in ΣSFA was observed with increasing dietary inclusion of microalgal oil, with the highest values recorded in fish fed the MO100 diet. In contrast, individual saturated fatty acids such as 14:0, 16:0, and 18:0 did not show differences among dietary treatments. Regarding monounsaturated fatty acids, oleic acid (18:1n‐9) decreased with increasing dietary inclusion of microalgal oil. In contrast, 18:1n‐7 increased in fish fed the MO100 diet compared with the other dietary treatments. Overall, ΣMUFA also decreased as increased dietary inclusion of microalgal oil, with significant differences observed between the MO50 and MO100 batches.

**Table 7:** Muscle fatty acid (% total fatty acids) profile and carotenoid (µg kg^−1^) content in gilthead seabream juveniles fed the experimental diets.

Fatty acid	MO50	MO75	MO100	*p*‐Value
14:0	2.4 ± 0.1	2.3 ± 0.0	2.2 ± 0.0	0.0729
16:0	17.0 ± 0.1	17.8 ± 0.1	17.4 ± 0.1	0.3747
18:0	3.9 ± 0.1	3.8 ± 0.1	3.9 ± 0.1	0.5896
16:1n‐7	5.8 ± 0.5	5.9 ± 0.1	5.8 ± 0.4	0.9614
18:1n‐9	26.7 ± 0.9^b^	25.0 ± 0.4^b^	23.0 ± 1.0^a^	0.0055
18:1n‐7	0.5 ± 0.0^a^	0.5 ± 0.0^a^	0.7 ± 0.1^b^	0.0234
18:2n‐6	12.8 ± 0.2^b^	13.1 ± 0.2^b^	11.7 ± 0.4^a^	0.0075
20:4n‐6, ARA	1.5 ± 0.2^a^	1.2 ± 0.1^a^	2.6 ± 0.4^b^	0.0265
18:3n‐3	2.7 ± 0.1	2.6 ± 0.0	2.7 ± 0.1	0.6114
18:4n‐3	0.7 ± 0.0^c^	0.6 ± 0.0^b^	0.5 ± 0.1^a^	0.0010
20:4n‐3	1.9 ± 0.1	1.6 ± 0.0	1.8 ± 0.2	0.1547
20:5n‐3, EPA	6.0 ± 0.0^a^	6.6 ± 0.0^b^	7.1 ± 0.1^c^	<0.0001
22:5n‐3	0.7 ± 0.0	0.5 ± 0.0	0.7 ± 0.1	0.3978
22:6n‐3, DHA	16.5 ± 0.2	16.5 ± 0.2	16.6 ± 0.2	0.9575
ΣSFA	22.7 ± 0.1^a^	23.9 ± 0.1^b^	24.3 ± 0.1^c^	0.0150
ΣMUFA	32.5 ± 0.2^b^	31.7 ± 0.4^ab^	30.2 ± 0.4^a^	0.0123
n‐3	29.1 ± 0.3	28.7 ± 0.4	29.2 ± 0.3	0.1812
n‐6	14.4 ± 0.2	14.4 ± 0.2	14.6 ± 0.3	0.5206
n‐3/n‐6	2.0 ± 0.0	2.3 ± 0.0	2.0 ± 0.1	0.5567
EPA/DHA	0.4 ± 0.0^b^	0.4 ± 0.0^a^	0.4 ± 0.0^a^	0.2739
Anteroxanthin	nd	4.57 ± 0.1^b^	3.69 ± 0.0^a^	0.0488
Vaucheroxanthin	9.36 ± 0.92^b^	8.68 ± 0.3^b^	5.51 ± 0.0^a^	0.0261
Vaucheroxanthin ester	8.72 ± 0.3	9.65 ± 0.1	8.84 ± 0.2	0.6853
Cantaxanthin	5.32 ± 0.2^b^	3.61 ± 0.1^a^	2.06 ± 0.0^a^	0.0291
*β*‐carotene	41.72 ± 0.8^ab^	38.92 ± 0.8^a^	46.32 ± 0.8^b^	0.0082
Total carotenoids	63.67 ± 0.8	62.85 ± 1.4	63.87 ± 0.8	0.8873

*Note:* MO50: diet with 50% microalgal oil; MO75: diet with 75% microalgal oil; MO100: diet with 100% microalgal oil. Values are expressed as mean ± SEM (*n* = 3). Values in the same row with different lowercase letters indicate significant difference among dietary treatments (*p* < 0.05).

Abbreviations: ARA, arachidonic acid; DHA, docosahexaenoic acid; EPA, eicosapentaenoic acid; MUFA, monounsaturated fatty acids; nd, not detected; PUFA, polyunsaturated fatty acids; SFA, saturated fatty acids.

For polyunsaturated fatty acids, 18:2n‐6 decreased in fish fed the MO100 diet compared with MO50 and MO75, whereas 20:4n‐6 (ARA) increased in MO100 fish. The intermediate fatty acid 18:4n‐3 decreased progressively with increasing microalgal oil inclusion. EPA (20:5n‐3) increased across treatments, reaching the highest values in MO100 fish, while DHA (22:6n‐3) remained unaffected by dietary treatment. Consequently, no differences were observed in total n‐3, total n‐6, n‐3/n‐6 ratio, or EPA/DHA ratio among experimental groups. In the case of polyunsaturated fatty acids, linoleic acid (18:2n‐6) decreased in fish fed the MO100 diet compared with MO50 and MO75, whereas arachidonic acid (20:4n‐6, ARA) increased in individuals from the MO100 group. Stearidonic acid (SDA; 18:4n‐3) showed a progressive decrease as dietary inclusion of microalgal oil increased (Supporting Information 1: Table S1). EPA (20:5n‐3) reached the highest values in MO100, whereas DHA (22:6n‐3) was not affected by dietary treatment. Consequently, no differences were observed in total n‐3 or n‐6 content, nor in the n‐3/n‐6 and EPA/DHA ratios among experimental groups.

Carotenoid composition was also affected by dietary treatment (Table 7). Vaucheroxanthin decreased significantly with increasing inclusion of microalgal oil, with the lowest values observed in fish fed the MO100 diet. In contrast, *β*‐carotene showed a significant increase in MO100 compared with MO75, with intermediate values in MO50. Total carotenoid content did not differ among dietary treatments. Anteroxanthin was not detected in fish fed the MO50 diet, whereas it was present in fish fed MO75 and MO100, with higher values observed in MO75. Cantaxanthin decreased significantly with increasing dietary inclusion of microalgal oil, with the highest values recorded in MO50 and the lowest in MO100. Vaucheroxanthin ester did not differ among experimental groups.

### 3.4. Blood and Tissue Biochemistry

Plasma, hepatic, and muscle metabolite concentrations in gilthead seabream juveniles fed the different experimental diets are presented in Table 8. In plasma, only triglyceride concentration was significantly affected by the dietary treatment, showing a progressive decrease with increasing levels of microalgal oil inclusion. Fish fed the MO100 diet exhibited significantly lower plasma triglyceride concentrations than those fed the MO50 diet, whereas the MO75 group showed intermediate values, with no significant differences from either of the other dietary treatments. In contrast, plasma glucose, lactate, protein, cholesterol, and cortisol concentrations did not differ significantly among the dietary treatments, although plasma glucose showed a tendency to increase in the MO100 group without reaching statistical significance.

**Table 8:** Concentration of plasma, liver, and muscle metabolites in gilthead seabream juveniles fed the different experimental diets.

Metabolites	MO50	MO75	MO100	*p*‐Value
Plasma metabolites				
Glucose (mM)	3.8 ± 0.2	3.7 ± 0.1	4.4 ± 0.3	0.0755
Lactate (mM)	0.5 ± 0.0	0.5 ± 0.1	0.5 ± 0.0	0.9761
Triglycerides (mM)	2.9 ± 0.2^b^	2.3 ± 0.3^ab^	1.9 ± 0.3^a^	0.0004
Proteins (mg dL^−1^)	30.4 ± 0.8	30.0 ± 1.3	28.4 ± 1.6	0.5288
Cholesterol (mg dL^−1^)	67.6 ± 5.8	62.9 ± 4.6	66.7 ± 3.2	0.7506
Cortisol (ng mL^−1^)	8.4 ± 0.8	8.2 ± 0.7	8.1 ± 0.4	0.1701
Hepatic metabolites				
Lactate (mM)	0.5 ± 0.2	0.5 ± 0.1	0.6 ± 0.1	0.7339
Triglycerides (mM)	28.1 ± 6.7	35.7 ± 8.9	37.2 ± 6.3	0.6926
Glucose (mM)	8.2 ± 1.4	4.9 ± 1.2	9.4 ± 1.3	0.1893
Glycogen (mM)	36.1 ± 8.6	55.2 ± 11.1	42.3 ± 6.3	0.2580
Muscle metabolites				
Lactate (mM)	3.5 ± 0.3	3.7 ± 0.5	3.9 ± 0.3	0.8575
Triglycerides (mM)	6.5 ± 2.4	4.0 ± 1.2	2.9 ± 0.8	0.3980
Glucose (mM)	3.7 ± 0.8	3.5 ± 0.7	6.0 ± 0.9	0.1816
Glycogen (mM)	24.2 ± 4.4	28.2 ± 5.0	27.5 ± 1.9	0.8312

*Note:* MO50: diet with 50% microalgal oil; MO75: diet with 75% microalgal oil; MO100: diet with 100% microalgal oil. Values are expressed as mean ± SEM (*n* = 3). Values in the same row with different lowercase letters indicate significant difference among dietary treatments (*p* < 0.05).

In the liver and muscle, lactate, triglyceride, glucose, and glycogen concentrations were not affected by the level of microalgal oil inclusion in the diet.

### 3.5. Digestive Enzyme Activities

**Figure 1:**
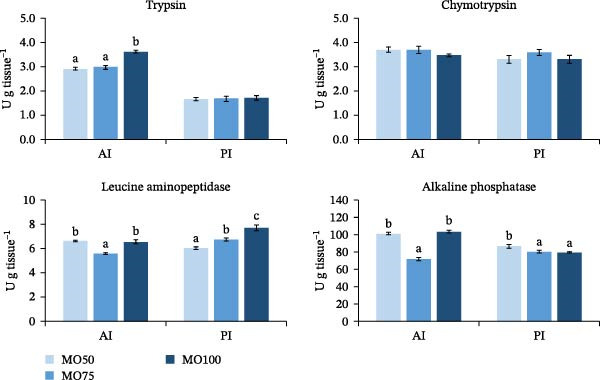
Digestive enzyme activities in anterior (AI) and posterior (PI) intestinal extracts in juvenile gilthead seabream fed the experimental diets. MO50: diet with 50% microalgal oil; MO75: diet with 75% microalgal oil; MO100: diet with 100% microalgal oil. Values are expressed as mean ± SEM (*n* = 3). Different lowercase letters indicate significant differences among dietary treatments (*p* < 0.05).

Figure 1 shows the activity level of the different digestive enzymes measured in the anterior and posterior intestinal sections of juvenile gilthead seabream fed the experimental diets. Trypsin activity in the anterior intestine was similar in fish fed the MO50 and MO75 groups, while significantly higher activity was observed in the MO100 group (*p* < 0.0001). In contrast, in the posterior intestine, trypsin activity showed no significant differences among the experimental groups. Chymotrypsin activity did not differ significantly in either the anterior or posterior intestinal sections across all treatments.

Intestinal enzyme activities exhibited clear differences associated with the type of diet provided. Leucine aminopeptidase activity in the anterior intestine was lower in the MO50 and MO100 groups compared with MO75 (*p* = 0.0111). In the posterior intestine, leucine aminopeptidase activity differed among all experimental diets, with MO50, MO75, and MO100 showing distinct values (*p* = 0.0080). Alkaline phosphatase activity in the anterior intestine was significantly higher in the MO50 and MO100 groups compared with MO75 (*p* = 0.0077). In contrast, in the posterior intestine, enzyme activity was higher in MO50 (*p* = 0.0090), while MO75 and MO100 were similar.

### 3.6. Intestinal Histology

**Figure 2:**
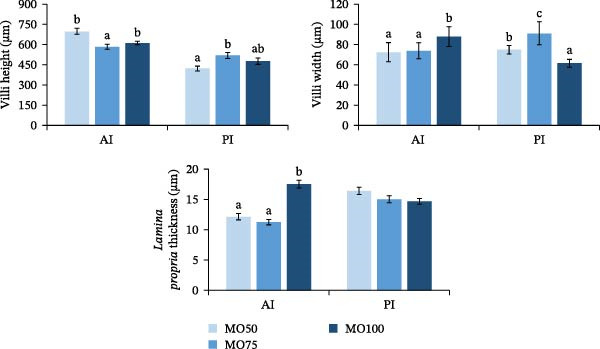
Histological parameters measured in the anterior (AI) and posterior (PI) intestine in juvenile gilthead seabream fed the different experimental diets. MO50: diet with 50% microalgal oil; MO75: diet with 75% microalgal oil; MO100: diet with 100% microalgal oil. Values are expressed as mean ± SEM (*n* = 6). Different lowercase letters indicate significant difference among dietary treatments (*p* < 0.05).

No signs of damage or diet‐related morphological alterations in the intestinal mucosa structure were detected (Supporting Information 2: Figure S1). Moreover, the arrangement of epithelial cells and overall tissue integrity appeared to be consistently maintained throughout the analyzed intestinal section. Figure 2 shows the results obtained of the histomorphological analysis obtained from light microscopy images of the anterior and posterior intestinal sections. In the anterior intestine, VH was significantly higher in MO50 and MO100 groups than in MO75 group. In the posterior intestine, the highest VH was observed in fish fed the MO75 diet differing significantly from the MO50 group. Regarding VW, in the anterior intestine, MO100 exhibited significantly higher values than MO50 and MO75, which did not differ from each other. In the posterior intestine, MO75 showed the highest VW, followed by MO50 and then MO100. LT in the anterior intestine was significantly reduced in MO100 compared with MO50 and MO75 which showed similar values. No significant differences were detected among treatments in the posterior intestine.

## 4. Discussion

The rapidly increasing demand for fishmeal and fish oil, largely driven by the expansion of aquaculture and their application across multiple industries, has led to the need to identify alternative sources capable of ensuring the long‐term sustainability and economic viability of the sector. In this context, microalgae‐derived products have emerged as a promising alternative due to their high nutritional value and the beneficial effects reported on fish growth, health, and overall performance [1, 2, 22]. Previous studies have demonstrated that microalgae can be used as a functional ingredient and as a partial or total substitute for fishmeal and fish oil, leading to improvements in fillet fatty acid profiles, metabolic activity, intestinal absorption, and overall performance in aquaculture species [2, 57, 58]. These findings highlight the importance of investigating the partial and total replacement of fish oil in aquaculture diets, including those for gilthead seabream (*Sparus aurata*).

The results obtained in the present study demonstrate that the different levels of dietary microalgal oil inclusion did not affect the growth performance or any of the zootechnical parameters evaluated. Similar outcomes were reported by Santigosa et al. [59] and Sarker et al. [19] when fish oil was replaced with microalgae oil in diets for gilthead seabream and Nile tilapia (*Oreochromis niloticus*), respectively. Comparable results have also been observed in other fish species, such as *Pagrus major* [60], *Oncorhynchus mykiss* [1], and *Salmo salar* [61], in which total replacement of fish oil with microalgal oil did not result in significant differences in growth performance or feed efficiency. Overall, the results obtained are consistent with those reported by Karapanagiotidis et al. [2] and Ayala et al. [62] following the inclusion of microalgae biomass in diets for juvenile gilthead seabream.

The proximate composition of the muscle provides information on fish quality, nutritional value, and physiological status. These parameters reflect available energy reserves, nutrient storage, and the ability of fish to adapt to their environment and diet [63, 64]. In the present study, the different levels of dietary microalgal oil inclusion did not affect the proximate composition of muscle, as no significant differences were observed in protein, lipid, moisture, or ash contents among the experimental treatments. These results are consistent with previous studies in gilthead seabream, in which the partial or complete replacement of fish oil with microalgal oil or microalgae‐derived products likewise did not result in significant changes in the proximate composition of the muscle or fillet. In particular, Karapanagiotidis et al. [2] and Carvalho et al. [65] demonstrated that the complete replacement of fish oil with microalgal oil‐based formulations maintained unchanged muscle protein, lipid, moisture, and ash contents, despite altering the fatty acid composition of the tissue, suggesting that the inclusion of microalgal oil preserves the nutritional quality of the muscle.

The absence of differences in muscle lipid content was accompanied by similar muscle and hepatic triglyceride concentrations among the dietary treatments, suggesting that increasing the level of dietary microalgal oil inclusion did not alter lipid storage in these tissues. Although plasma triglyceride concentrations decreased with increasing levels of microalgal oil inclusion, this response was not reflected in either the total muscle lipid content or tissue triglyceride concentrations [21, 66, 67]. Since plasma triglycerides primarily reflect lipid transport in circulation rather than lipid deposition within tissues, the present results do not allow a direct relationship to be established between these responses.

Beyond total lipid content, the determination of the muscle fatty acid profile is of great importance, as it enables not only the assessment of fillet nutritional value for human consumption but also a better understanding of how dietary composition influences lipid metabolism in fish [68–70]. In this context, various microalgae species are characterized by a high content of essential fatty acids, which play a fundamental role in fish growth, health maintenance, and metabolic regulation [19, 71, 72]. The incorporation of microalgae into aquaculture diets not only helps to meet the nutritional requirements of cultured species but also contributes to the production of final products with an improved nutritional and functional profile, thereby enhancing their value both from a production standpoint and for human consumption [16, 26, 72].

In this study, fish oil was replaced by a blend of *M. gaditana* and *I. galbana* oils containing, among others, EPA, DHA, and 18:4n‐3 (SDA). The progressive inclusion of this microalgal oil blend significantly modified the muscle fatty acid profile, as evidenced by an increase in EPA content. This finding was consistent with the higher dietary supply of this fatty acid and agrees with previous reports in gilthead seabream, olive flounder (*Paralichthys olivaceus*), and totoaba (*Totoaba macdonaldi*), in which the replacement of fish oil with microalgal oil resulted in increased tissue deposition of EPA without compromising DHA content [2, 20, 73]. In addition to the increase in EPA, dietary microalgal oil modified the relative proportions of saturated and monounsaturated fatty acids in muscle, with increasing SFA and decreasing MUFA contents as the level of fish oil replacement increased. These changes closely reflected the fatty acid composition of the experimental diets, suggesting a direct dietary influence on tissue fatty acid deposition. Similar responses have been reported in gilthead seabream fed microalgal oils, indicating that dietary fatty acid composition is readily mirrored in muscle lipids, particularly for SFA and MUFA [2, 65].

Despite the high 18:4n‐3 (SDA) content of the microalgal oil blend, mainly contributed by *I. galbana* [74], this fatty acid accounted for only 0.3%–0.4% of the total fatty acids in the experimental diets, and its muscle content decreased as the level of dietary microalgal oil inclusion increased. The relatively low proportion of 18:4n‐3 in the diets can be explained, at least in part, by the diet formulation, as the microalgal oil constituted only a fraction of the total lipid content and was combined with other lipid sources. In addition, some degradation during the extrusion process cannot be ruled out since polyunsaturated fatty acids are particularly susceptible to thermal and oxidative degradation [75]. However, the relative contribution of these two factors cannot be determined from the available data and would require dedicated studies evaluating the stability of 18:4n‐3 during feed manufacture.

Carotenoid deposition in fish tissues is influenced by a combination of dietary, physiological, and species‐specific factors, including the chemical structure, bioavailability, absorption, transport, and tissue retention of individual carotenoids [76, 77]. Owing to their well‐established antioxidant and anti‐inflammatory properties, carotenoids play an important role in protecting tissues against oxidative damage by scavenging free radicals and reactive oxygen species (ROS), thereby contributing to the maintenance of cellular homeostasis [76, 77]. In the present study, increasing the dietary inclusion of microalgal oil did not affect the total carotenoid content of the muscle, indicating that the overall carotenoid deposition remained unchanged among the experimental treatments. However, significant differences were observed in the concentrations of individual carotenoids, including antheraxanthin, vaucheroxanthin, canthaxanthin, and *β*‐carotene, demonstrating that dietary lipid composition influenced the muscle carotenoid profile rather than the total amount accumulated. Although all experimental diets contained the same level of microalgal carotenoid extract, slight differences in their carotenoid composition, together with the progressive replacement of fish oil by microalgal oil, may have affected the bioavailability and tissue deposition of specific carotenoids. These findings suggest that the deposition of individual carotenoids is not solely determined by their dietary concentration but also by selective processes governing their absorption, transport, metabolism, and retention within the muscle. Similar observations have previously been reported in fish, where carotenoid accumulation was shown to depend not only on dietary supply but also on the physicochemical properties of each carotenoid and its interaction with dietary lipids [22, 78–80].

The evaluation of metabolites in plasma, liver, and muscle constitutes a valuable approach for assessing the metabolic status and physiological responses of fish to nutritional changes [81, 82]. In the present study, the absence of significant differences in plasma glucose, glycogen, lactate, protein, cholesterol, and cortisol concentrations, as well as in hepatic and muscle metabolites, indicates that the progressive replacement of fish oil with microalgal oil did not alter the overall metabolic status of juvenile gilthead seabream. These findings are consistent with previous studies reporting that the replacement of fish oil with microalgal oil did not affect growth performance, feed utilization, or other physiological indicators when an adequate supply of long‐chain n‐3 fatty acids was maintained [2, 59, 83].

The only plasma metabolite affected by the dietary treatments was triglyceride concentration, which decreased progressively with increasing dietary microalgal oil inclusion. However, this reduction was not accompanied by changes in hepatic or muscle triglyceride concentrations or in muscle lipid content, suggesting that it reflects alterations in systemic lipid metabolism or circulating lipid transport rather than changes in tissue lipid storage.

Digestive capacity of fish constitutes a fundamental pillar for the efficient utilization of nutrients and the maintenance of zootechnical performance [84–86]. Assessing digestive functionality, including digestive enzyme activity and the integrity of intestinal mucosa, provides valuable insight into the physiological adaptation of fish to changes in dietary composition [87, 88]. This aspect is particularly relevant when introducing alternative ingredients such as microalgal oils and biomasses, whose effects on the digestion and absorption of macronutrients may influence not only growth efficiency but also intestinal health and overall animal welfare [89, 90].

In the present study, the progressive replacement of fish oil with microalgal oil modified the activity of several digestive enzymes, although these responses depended on both the enzyme evaluated and the intestinal segment considered. Trypsin activity was affected only in the anterior intestine, where fish fed the MO100 diet exhibited higher activity than those fed the MO50 and MO75 diets, whereas no differences were detected in the posterior intestine. By contrast, chymotrypsin activity remained unchanged in both intestinal segments, indicating that fish oil replacement did not generally affect protein digestion. These findings are consistent with previous studies reporting that the inclusion of microalgal oils or other microalgae‐derived ingredients can selectively modulate the activity of digestive enzymes without compromising growth performance or feed utilization, reflecting the adaptive capacity of the digestive system to changes in dietary composition [91–93].

The responses observed for leucine aminopeptidase and alkaline phosphatase were more dependent on the intestinal region. In the anterior intestine, leucine aminopeptidase activity was higher in fish fed the MO75 diet than in those receiving the MO50 and MO100 diets, whereas significant differences among all three dietary treatments were observed in the posterior intestine. Likewise, alkaline phosphatase activity exhibited contrasting patterns between intestinal segments, being higher in MO50 and MO100 than in MO75 in the anterior intestine, while in the posterior intestine the lowest activity was recorded in fish fed the MO50 diet. Alkaline phosphatase is involved in intestinal digestion and absorption and is widely recognized as a marker of brush‐border functionality and enterocyte maturation [94, 95]. However, since the enzymatic changes observed were not accompanied by alterations in growth performance, body composition, or metabolic status, they are more likely to reflect physiological adjustments of the intestinal epithelium to differences in dietary lipid composition than impairment of digestive capacity.

Histological examination of the intestine complemented the enzymatic analyses by determining whether the observed changes in enzyme activity were associated with structural modifications of the intestinal mucosa. Regardless of the dietary treatment, the overall architecture of the intestinal epithelium remained well preserved, and no lesions, inflammatory signs, or morphological alterations attributable to fish oil replacement were detected. Likewise, the organization of enterocytes and the integrity of the intestinal mucosa were maintained in all experimental groups, indicating that dietary microalgal oil did not compromise intestinal structural integrity. These findings agree with previous studies in gilthead seabream showing that the inclusion of microalgae‐derived ingredients does not adversely affect intestinal morphology [12, 56].

Although no histopathological alterations were detected, histomorphometric analysis revealed diet‐dependent changes in several intestinal parameters. In the anterior intestine, fish fed the MO50 and MO100 diets exhibited greater VH than those fed the MO75 diet, whereas VW and LT were the highest in the MO100 group. In contrast, the posterior intestine displayed a different response pattern, with the greatest VH observed in fish fed the MO75 diet, while the VW increased progressively from MO75 to MO100. Previous studies have shown that increased VH and VW are generally associated with a greater potential absorptive surface and are considered indicators of preserved intestinal functionality [96–98]. Nevertheless, as nutrient absorption was not directly assessed in the present study, these findings should be interpreted with caution and regarded as morphological responses compatible with an adequate adaptation of the intestinal epithelium to the replacement of fish oil by microalgal oil.

The increase in LT observed in the anterior intestine of fish fed the MO100 diet was likewise not accompanied by histopathological lesions or changes in growth performance or metabolic parameters, making it difficult to associate this response with inflammatory processes. The *lamina propria* is a dynamic compartment composed of connective tissue, blood vessels, and immune cells, and its morphology may vary as a consequence of physiological tissue remodeling [95, 99]. In the absence of specific inflammatory or immune markers, the functional significance of this increase cannot be established from the present data, and further studies are required to elucidate the mechanisms involved.

## 5. Conclusion

The progressive replacement of fish oil with microalgal oil proved to be a suitable nutritional strategy for juvenile gilthead seabream (*Sparus aurata*). Increasing the dietary inclusion of microalgal oil from 50% to 100% of the added oil fraction did not adversely affect growth performance, biometric indices, proximal composition, metabolic status, digestive functionality, or intestinal integrity. Furthermore, dietary microalgal oil modified the muscle fatty acid profile by increasing EPA deposition while maintaining DHA levels without altering the overall nutritional quality of the muscle. It also induced changes in the muscle carotenoid profile and in selected digestive enzyme activities and intestinal histomorphometric parameters, indicating an adaptive response of the digestive tract without evidence of functional impairment. Overall, these findings support the use of microalgal oil as a sustainable alternative to fish oil in diets for juvenile gilthead seabream. Future studies should evaluate the long‐term effects of this nutritional strategy and its applicability to other marine carnivorous species of aquaculture interest, such as the European seabass and greater amberjack.

## Author Contributions

**Sara Flores-Moreno**: experimentation, formal analysis, writing – original draft, writing – review and editing. **Alba Galafat**: data curation, writing – review and editing. **Antonio J. Vizcaíno and Alejandro Blázquez-Durán**: methodology. **Verónica de las Heras and Marta Román**: experimentation. **Paula Simó-Mirabet**: methodology, data curation. **Ismael Hachero-Cruzado and Elvira Navarro-López**: formal analysis. **Miguel Angel González-Cardoso**: methodology. **Mari Carmen Cerón-García**: funding acquisition, conceptualization. **Juan Antonio Martos-Sitcha**: conceptualization, writing – review and editing. **Francisco Javier Alarcón-López**: funding acquisition, conceptualization, writing – review and editing.

## Funding

This study was funded by the project UAL2020‐TEP‐A2001 from the UALFEDER 2020 call and the CEI·MAR Research Project granted to the spin‐off LifeBioencapsulation S.L. (Convocatoria de Proyectos de Innovación Empresarial con Proyección Territorial 2022). Sara Flores contract is supported by the European Union’s Horizon 2020 research and innovation programme (Grant 101036768) (NeoGiANT project). This work was supported by the project “ULVACTIVE” (Grant PID2023‐152514OB‐C21), funded by the Spanish MCIU.

## Conflicts of Interest

The authors declare no conflicts of interest.

## Supporting Information

Additional supporting information can be found online in the Supporting Information section.

## Supporting information


**Supporting Information 1** Table S1: Linear regression analysis (*R* ^2^ and *p*‐values) of muscle fatty acid profiles in gilthead seabream fed experimental diets.

**Supporting Information 2** Figure S1: Details on microphotographs of the anterior and posterior intestine in juvenile gilthead seabream fed the experimental diets.

## Data Availability

Data will be made available upon request.

## References

[1] Sarker P. K. , Kapuscinski A. R. , McKuin B. , Fitzgerald D. S. , Nash H. M. , and Greenwood C. , Microalgae-Blend Tilapia Feed Eliminates Fishmeal and Fish Oil, Improves Growth, and Is Cost Viable, Scientific Reports. (2020) 10, no. 1, 10.1038/s41598-020-75289-x, 19328. 33184333 PMC7665073

[2] Karapanagiotidis I. T. , Metsoviti M. N. , and Gkalogianni E. Z. , et al.The Effects of Replacing Fish Meal With *Chlorella vulgaris* and Fish Oil With *Schizochytrium* sp. and *Microchloropsis gaditana* Blend on Growth Performance, Feed Efficiency, Muscle Fatty Acid Composition, and Liver Histology of Gilthead Seabream (*Sparus aurata*), Aquaculture. (2022) 561, 10.1016/j.aquaculture.2022.738709, 738709.

[3] Cashion T. , Le Manach F. , Zeller D. , and Pauly D. , Most Fish Destined for Fishmeal Production are Food-Grade Fish, Fish and Fisheries. (2017) 18, no. 5, 837–844, 10.1111/faf.12209.

[4] Gasco L. , Gai F. , and Maricchiolo G. , et al.Fishmeal Alternative Protein Sources for Aquaculture Feeds, Feeds for the Aquaculture Sector. (2018) 15, no. 16, 10.1007/978-3-319-77941-6, 12500.

[5] Cottrell R. S. , Blanchard J. L. , Halpern B. S. , Metian M. , and Froehlich H. E. , Global Adoption of Novel Aquaculture Feeds Could Substantially Reduce Forage Fish Demand by 2030, Nature Food. (2020) 1, no. 5, 301–308, 10.1038/s43016-020-0078-x.

[6] McKuin B. , Kapuscinski A. R. , Sarker P. K. , Cheek N. , Lim J. , and Sabarsky M. , Comparative Life Cycle Assessment of Marine Microalgae, *Nannochloropsis* sp. and Fishmeal for Sustainable Protein Ingredients in Aquaculture Feeds, Elementa Science of the Anthopocene. (2023) 11, no. 1, 10.1525/elementa.2022.00083, 00083.

[7] Sarker M. M. , Khan S. , Haque M. M. , Shahjahan M. , Begum M. , and Reza M. S. , Unraveling the Nutritional Potential of the Microalga *Scenedesmus* sp. and the Zooplankton *Diaphanosoma* sp. as Initial Foods for the Rearing of Rohu (*Labeo rohita*) Larvae, Aquaculture International. (2025) 33, no. 6, 10.1007/s10499-025-02106-5.

[8] Ahmad A. W. , Hassan S. , and Banat F. , An Overview of Microalgae Biomass as a Sustainable Aquaculture Feed Ingredient: Food Security and Circular Economy, Bioengineered. (2022) 13, no. 4, 9521–9547, 10.1080/21655979.2022.2061148. 35387561 PMC9161971

[9] Tham P. E. , Lim H. R. , and Khoo K. S. , et al.Insights of Microalgae-Based Aquaculture Feed: A Review on Circular Bioeconomy and Perspectives, Algal Research. (2023) 74, 10.1016/j.algal.2023.103186, 103186.

[10] Gao S. , Chen W. , Cao S. , Sun P. , and Gao X. , Microalgae as Fishmeal Alternatives in Aquaculture: Current Status, Existing Problems, and Possible Solutions, Environmental Science and Pollution Research. (2024) 31, no. 11, 16113–16130, 10.1007/s11356-024-32143-1. 38315337

[11] Chen C. Z. , Li P. , Liu L. , and Li Z. H. , Exploring the Interactions Between the Gut Microbiome and the Shifting Surrounding Aquatic Environment in Fisheries and Aquaculture: A Review, Environmental Research. (2022) 214, 10.1016/j.envres.2022.114202, 114202. 36030922

[12] Galafat A. , Vizcaíno A. J. , and Sáez M. I. , et al.Assessment of Dietary Inclusion of Crude or Hydrolysed *Arthrospira platensis* Biomass in Starter Diets for Gilthead Seabream (*Sparus aurata*), Aquaculture. (2022) 548, 10.1016/j.aquaculture.2021.737680, 737680.

[13] Dineshbabu G. , Goswami G. , Kumar R. , Sinha A. , and Das D. , Microalgae–Nutritious, Sustainable Aqua-and Animal Feed Source, Journal of Functional Foods. (2019) 62, 10.1016/j.jff.2019.103545, 103545.

[14] Andreeva A. , Budenkova E. , and Babich O. , et al.Production, Purification, and Study of the Amino Acid Composition of Microalgae Proteins, Molecules. (2021) 26, no. 9, 10.3390/molecules26092767, 2767. 34066679 PMC8125830

[15] Shah M. R. , Lutzu G. A. , and Alam A. , et al.Microalgae in Aquafeeds for a Sustainable Aquaculture Industry, Journal of Applied Phycology. (2018) 30, no. 1, 197–213, 10.1007/s10811-017-1234-z.

[16] Ansari F. A. , Guldhe A. , Gupta S. K. , Rawat I. , and Bux F. , Improving the Feasibility of Aquaculture Feed by Using Microalgae, Environmental Science and Pollution Research. (2021) 28, no. 32, 43234–43257, 10.1007/s11356-021-14989-x. 34173144

[17] Adarme-Vega T. C. , Thomas-Hall S. R. , and Schenk P. M. , Towards Sustainable Sources for Omega-3 Fatty Acids Production, Current Opinion in Biotechnology. (2014) 26, 14–18, 10.1016/j.copbio.2013.08.003. 24607804

[18] Sun G. , Zhang X. , and Zhang F. , et al.Use Microalgae to Treat Coke Wastewater for Producing Biofuel: Influence of Phenol on Photosynthetic Properties and Intracellular Components of Microalgae, Chemosphere. (2024) 349, 10.1016/j.chemosphere.2023.140805, 140805. 38040255

[19] Sarker P. , Kapuscinski A. , Lanois A. , Livesey E. , Bernhard K. , and Coley M. , Towards Sustainable Aquafeeds: Complete Substitution of Fish Oil With Marine Microalga *Schizochytrium* sp. Improves Growth and Fatty Acid Deposition in Juvenile Nile Tilapia (*Oreochromis niloticus*), PLoS ONE. (2016) 11, no. 6, 10.1371/journal.pone.0156684, e0156684. 27258552 PMC4892564

[20] Maldonado-Othón C. , Pérez-Velázquez M. , Gatlin D. , and González-Félix M. , Replacement of Fish Oil by Soybean Oil and Microalgal Meals in Diets for *Totoaba macdonaldi* (Gilbert, 1890) Juveniles, Aquaculture. (2020) 529, 10.1016/j.aquaculture.2020.735705, 735705.

[21] Perera E. , Sánchez-Ruiz D. , and Sáez M. I. , et al.Low Dietary Inclusion of Nutraceuticals From Microalgae Improves Feed Efficiency and Modifies Intermediary Metabolisms in Gilthead Seabream (*Sparus aurata*), Scientific Reports. (2020) 10, no. 1, 10.1038/s41598-020-75693-3, 18676. 33122726 PMC7596551

[22] Sales A. , Galafat A. , and Vizcaíno J. , et al.Effects of Dietary Use of Two Lipid Extracts From the Microalga *Nannochloropsis gaditana* (Lubián, 1982) Alone and in Combination on Growth and Muscle Composition in Juvenile Gilthead Seabream, *Sparus aurata* , Algal Research. (2021) 53, 10.1016/j.algal.2020.102162, 102162.

[23] Falaise C. , François C. , and Travers M. A. , et al.Antimicrobial Compounds From Eukaryotic Microalgae Against Human Pathogens and Diseases in Aquaculture, Marine Drugs. (2016) 14, no. 9, 10.3390/md14090159. PMC5039530 27598176

[24] Mishra N. , Prasad S. M. , and Mishra N. , Influence of High Light Intensity and Nitrate Deprivation on Growth and Biochemical Composition of the Marine Microalgae *Isochrysis galbana* , Brazilian Archives of Biology and Technology. (2019) 62, 10.1590/1678-4324-2019180398, e19180398.

[25] Zhou J. , Wang M. , and Saraiva J. A. , et al.Extraction of Lipids From Microalgae Using Classical and Innovative Approaches, Food Chemistry. (2022) 384, 10.1016/j.foodchem.2022.132236, 132236. 35240572

[26] Mingyang M. and Hu Q. , Microalgae as Feed Sources and Feed Additives for Sustainable Aquaculture: Prospects and Challenges, Reviews in Aquaculture. (2023) 16, no. 2, 818–835, 10.1111/raq.12869.

[27] Soyocak H. , Kızılkaya D. , and Üstün N. , A Review about a Significant Source of Bioactive Compounds: Microalgae, International Journal of Innovative Approaches in Agricultural Research. (2023) 7, no. 3, 371–387, 10.29329/ijiaar.2023.602.11.

[28] Hu X. , Ma W. , and Zhang D. , et al.Application of Natural Antioxidants as Feed Additives in Aquaculture: A Review, Biology. (2025) 14, no. 1, 10.3390/biology14010087. PMC11762552 39857317

[29] Sales R. , Cerón-García M. C. , and Navarro-López E. , et al.Processing *Nannochloropsis gaditana* Biomass for the Extraction of High-Value Biocompounds, Journal of Applied Phycology. (2020) 32, no. 5, 3113–3122, 10.1007/s10811-020-02156-7.

[30] Monteiro M. , Lavrador A. S. , and Santos R. , et al.Evaluation of the Potential of Marine Algae Extracts as a Source of Functional Ingredients Using Zebrafish as Animal Model for Aquaculture, Marine Biotechnology. (2021) 23, no. 4, 529–545, 10.1007/s10126-021-10044-5. 34189658

[31] Cusimano G. , Cueto P. , and Lladó S. , et al.Effect of Water Salinity and Dietary Supplementation With *Microchloropsis gaditana* on Growth, Health, Quality and Microbiota of Barramundi *Lates calcarifer* , Open Research Europe. (2025) 5, 10.12688/openreseurope.18715.1.

[32] Díaz N. , Muñoz S. , Medina A. , Riquelme C. , and Lozano-Muñoz I. , *Microchloropsis gaditana* as a Natural Antimicrobial With a One Health Approach to Food Safety in Farmed Salmon, Life. (2025) 15, no. 3, 10.3390/life15030455. PMC11943575 40141798

[33] Camacho-Rodríguez J. , Cerón-García M. C. , González-López C. V. , López-Rosales L. , Contreras-Gómez A. , and Molina-Grima E. , Use of Continuous Culture to Develop an Economical Medium for the Mass Production of *Isochrysis galbana* for Aquaculture, Journal of Applied Phycology. (2020) 32, no. 2, 851–863, 10.1007/s10811-019-02015-0.

[34] Bustamam M. S. A. , Pantami H. A. , Azam A. A. , Shaari K. , Min C. C. , and Ismail I. S. , The Immunostimulant Effects of *Isochrysis galbana* Supplemented Diet on the Spleen of Red Hybrid Tilapia (*Oreochromis* spp.) Evaluated by Nuclear Magnetic Resonance Metabolomics, Aquaculture Nutrition. (2022) 2022, 10.1155/2022/1154558, 1154558.

[35] Wang C. , Jiang C. , and Gao T. , et al.Improvement of Fish Production and Water Quality in a Recirculating Aquaculture Pond Enhanced With Bacteria-Microalgae Association, Aquaculture. (2022) 547, 10.1016/j.aquaculture.2021.737420, 737420.

[36] Cao J. Y. , Kong Z. Y. , and Ye M. W. , et al.Metabolomic and Transcriptomic Analyses Reveal the Effects of Ultraviolet Radiation Deprivation on *Isochrysis galbana* at High Temperature, Algal Research. (2019) 38, 10.1016/j.algal.2019.101424, 101424.

[37] de Los Reyes C. , Ortega M. J. , Rodríguez-Luna A. , Talero E. , Motilva V. , and Zubia E. , Molecular Characterization and Anti-Inflammatory Activity of Galactosylglycerides and Galactosylceramides From the Microalga *Isochrysis galbana* , Journal of Agricultural and Food Chemistry. (2016) 64, no. 46, 8783–8794, 10.1021/acs.jafc.6b03931. 27786470

[38] Becker E. W. , Micro-Algae as a Source of Protein, Biotechnology Advances. (2007) 25, no. 2, 207–210, 10.1016/j.biotechadv.2006.11.002. 17196357

[39] Karkos P. D. , Leong S. C. , Karkos C. D. , Sivaji N. , and Assimakopoulos D. A. , Spirulina in Clinical Practice: Evidence-Based Human Applications, Evidence-Based Complementary and Alternative Medicine. (2011) 2011, no. 1, 10.1093/ecam/nen058, 531053. 18955364 PMC3136577

[40] Galafat A. , Vizcaíno A. J. , and Sáez M. I. , et al.Evaluation of *Arthrospira* sp. Enzyme Hydrolysate as Dietary Additive in Gilthead Seabream (*Sparus aurata*) Juveniles, Journal of Applied Phycology. (2020) 32, no. 5, 3089–3100, 10.1007/s10811-020-02141-0.

[41] Akbarbaglu Z. , Ayaseh A. , Ghanbarzadeh B. , Sarabandi K. , Kharazmi M. S. , and Jafari S. M. , Chemical Structure and Bio-Functional Properties of *Arthrospira platensis* Peptides Produced by Ultrasonication-Enzymolysis: Their Emulsification Capabilities, Process Biochemistry. (2023) 132, 191–199, 10.1016/j.procbio.2023.07.019.

[42] Spínola M. P. , Costa M. M. , and Tavares B. , et al.Impact of Long-Term Feeding a High Level of *Spirulina* Combined With Enzymes on Growth Performance, Carcass Traits and Meat Quality in Broiler Chickens, Frontiers in Veterinary Science. (2024) 11, 10.3389/fvets.2024.1451516, 1451516. 39257638 PMC11385295

[43] Srichanun M. , Tantikitti C. , Kortner T. M. , Krogdahl Å , and Chotikachinda R. , Effects of Different Protein Hydrolysate Products and Levels on Growth, Survival Rate and Digestive Capacity in Asian Seabass (*Lates calcarifer* Bloch) Larvae, Aquaculture. (2014) 428, 195–202, 10.1016/j.aquaculture.2014.03.004.

[44] Soriano-Jerez Y. , Gallardo-Rodríguez J. J. , and López-Rosales L. , et al.Preventing Biofouling in Microalgal Photobioreactors, Bioresource Technology. (2024) 407, 10.1016/j.biortech.2024.131125, 131125. 39025371

[45] González-Cardoso M. A. , Yepez R. , Navarro-López E. , Contreras-Gómez A. , Alarcón-López F. J. , and Cerón-García M. C. , Evaluation of Pretreatments Used to Obtain Hydrolysates From Microalgae Biomass, and Their Effects on the Recovery of Carotenoids and Fatty Acids Intended for Agriculture Applications, Algal Research. (2026) 94, 10.1016/j.algal.2025.104500, 104500.

[46] AOAC , Official Methods of Analysis, 2000, The Association of Official Analytical Chemisths.

[47] Folch J. , Lee M. , and Sloane-Stanley G. H. , A Simple Method for the Isolation and Purification of Total Lipids From Animal Tissues, Journal of Biological Chemistry. (1957) 226, no. 1, 497–509, 10.1016/S0021-9258(18)64849-5. 13428781

[48] Rodríguez-Ruiz J. , Belarbi E. H. , Sanchez JLG. , and Alonso D. L. , Rapid Simultaneous Lipid Extraction and Transesterification for Fatty Acid Analyses, Biotechnology Techniques. (1998) 12, no. 9, 689–691, 10.1023/A:1008812904017.

[49] Lepage G. and Roy C. C. , Improved Recovery of Fatty Acid Through Direct Transesterification Without Prior Extraction or Purification, Journal of Lipid Research. (1984) 25, no. 12, 1391–1396, 10.1016/S0022-2275(20)34457-6. 6530596

[50] Cerón-García M. C. , González-López C. V. , Camacho-Rodríguez J. , López-Rosales L. , García-Camacho F. , and Molina-Grima E. , Maximizing Carotenoid Extraction From Microalgae Used as Food Additives and Determined by Liquid Chromatography (HPLC), Food Chemistry. (2018) 257, 316–324, 10.1016/j.foodchem.2018.02.154. 29622217

[51] Keppler D. and Decker K. , Glycogen Determination With Amyloglucosidase, Methods of Enzymatic Analysis, 1974, 4, Academic Press, 1127–1131.

[52] Erlanger B. F. , Kokowsky N. , and Cohen W. , The Preparation and Properties of Two New Chromogenic Substrates of Trypsin, Archives of Biochemistry Biophyics. (1961) 95, no. 2, 271–278, 10.1016/0003-9861(61)90145-X. 13890599

[53] DelMar E. G. , Largman C. , Brodrick J. W. , and Geokas M. C. , A Sensitive New Substrate for Chymotrypsin, Analytical Biochemistry. (1979) 99, no. 2, 316–320, 10.1016/S0003-2697(79)80013-5. 574722

[54] Pfleiderer G. , Particle-Bound Aminopeptidase From Pig Kidney, Methods in Enzymology. (1970) 19, 514–521, 10.1016/0076-6879(70)19038-0.

[55] Bergmeyer H. U. , Methods of Enzymatic Analysis, 1974, Verlag Chemie-Academic Press.

[56] Vizcaíno A. J. , López G. , and Sáez M. I. , et al.Effects of the Microalga *Scenedesmus Almeriensis* as Fishmeal Alternative in Diets for Gilthead Sea Bream, *Sparus aurata*, Juveniles, Aquaculture. (2014) 431, 34–43, 10.1016/j.aquaculture.2014.05.010.

[57] Sarker P. K. , Kaouscinski A. R. , and Bae A. Y. , et al.Towards Sustainable Aquafeeds: Evaluating Substitution of Fishmeal With Lipid Extracted Microalgal Co-Product (*Nannochloropsis oculata*) in Diets of Juvenile Nile Tilapia (*Oreochromis niloticus*), PLoS One. (2018) 13, no. 7, 10.1371/journal.pone.0201315, e0201315. 30063730 PMC6067735

[58] García-Márquez J. , Vizcaíno A. J. , and Barany A. , et al.Evaluation of the Combined Administration of *Chlorella fusca* and *Vibrio proteolyticus* in Diets for *Chelon labrosus*: Effects on Growth, Metabolism, and Digestive Functionality, Animals. (2023) 13, no. 4, 10.3390/ani13040589. PMC9951767 36830376

[59] Santigosa E. , Brambilla F. , and Milanese L. , Microalgae Oil as an Effective Alternative Source of EPA and DHA for Gilthead Seabream (*Sparus aurata*) Aquaculture, Animals. (2021) 11, no. 4, 10.3390/ani11040971. PMC8065835 33807244

[60] Seong T. , Uno Y. , Kitagima R. , Kabeya N. , Haga Y. , and Satoh S. , Microalgae as Main Ingredient for Fish Feed: Non-Fish Meal and Non-Fish Oil Diet Development for Red Sea Bream, *Pagrus Major*, by Blending of Microalgae *Nannochloropsis, Chlorella* and *Schizochytrium* , Aquaculture Research. (2021) 52, no. 12, 6025–6036, 10.1111/are.15463.

[61] Miller M. R. , Nichols P. D. , and Carter C. G. , Replacement of Fish Oil With Thraustochytrid *Schizochytrium* sp. L Oil in Atlantic Salmon Parr (*Salmo salar* L) Diets, Comparative Biochemistry and Physiology Part A: Molecular & Integrative Physiology. (2007) 148, no. 2, 382–392, 10.1016/j.cbpa.2007.05.018. 17588797

[62] Ayala M. D. , Balsalobre N. , and Chaves-Pozo E. , et al.Long-Term Effects of a Short Juvenile Feeding Period With Diets Enriched With the Microalgae *Nannochloropsis gaditana* on the Subsequent Body and Muscle Growth of Gilthead Seabream, *Sparus aurata* L, Animals. (2023) 13, no. 3, 10.3390/ani13030482. PMC9913079 36766372

[63] Ahmed I. , Jan K. , Fatma S. , and Dawood M. A. , Muscle Proximate Composition of Various Food Fish Species and Their Nutritional Significance: A Review, Journal of Animal Physiology and Animal Nutrition. (2022) 106, no. 3, 690–719, 10.1111/jpn.13711. 35395107

[64] Chakraborty S. and Kausor M. , Nutrient Status of Some Food Fishes With Respect to Their Proximate Composition: A Review, Uttar Pradesh Journal Of Zoology. (2024) 45, no. 17, 707–722, 10.56557/upjoz/2024/v45i174416.

[65] Carvalho M. , Ginés R. , and Martín I. , et al.Genetic Selection for High Growth Improves the Efficiency of Gilthead Sea Bream (*Sparus aurata*) in Using Novel Diets With Insect Meal, Single-Cell Protein and a DHA Rich-Microalgal Oil, Aquaculture. (2023) 578, 10.1016/j.aquaculture.2023.740034, 740034.

[66] Guerreiro I. , Magalhães R. , and Coutinho F. , et al.Evaluation of the Seaweeds *Chondrus crispus* and *Ulva lactuca* as Functional Ingredients in Gilthead Seabream (*Sparus aurata*), Journal of Applied Phycology. (2019) 31, no. 3, 2115–2124, 10.1007/s10811-018-1708-7.

[67] Antunes M. , Neves M. , and Pires D. , et al.Proximate Composition and Fatty Acid Profile of Gilthead Seabream (*Sparus aurata*) Fed With *Pelvetia canaliculata*-Supplemented Diets: An Insight Towards the Valorization of Seaweed Biomass, Foods. (2023) 12, no. 9, 10.3390/foods12091810, 1810. 37174348 PMC10178326

[68] Qiu H. , Jin M. , Li Y. , Lu Y. , Hou Y. , and Zhou Q. , Dietary Lipid Sources Influence Fatty Acid Composition in Tissue of Large Yellow Croaker (*Larmichthys crocea*) by Regulating Triacylglycerol Synthesis and Catabolism at the Transcriptional Level, PLoS ONE. (2017) 12, no. 1, 10.1371/journal.pone.0169985, e0169985. 28081221 PMC5231348

[69] He L. , Qin Y. , Wang Y. , Li D. , Chen W. , and Ye J. , Effects of Dietary Replacement of Fish Oil With Soybean Oil on the Growth Performance, Plasma Components, Fatty Acid Composition and Lipid Metabolism of Groupers *Epinephelus coioides* , Aquaculture Nutrition. (2021) 27, no. 5, 1494–1511, 10.1111/anu.13292.

[70] Yuan X. , Liu R. , Wei M. , Li H. , Sun J. , and Ji H. , Fish Oil Replacement With Different Vegetable Oils in *Onychostoma macrolepis*: Effects on Fatty Acid Metabolism Based on Whole-Body Fatty Acid Balance Method and Genes Expression, Fish Physiology and Biochemistry. (2024) 50, no. 4, 1583–1603, 10.1007/s10695-024-01357-y. 38739220

[71] Li S. , Wang B. , and Liu L. , et al.Enhanced Growth Performance Physiological and Biochemical Indexes of *Trachinotus ovatus* Fed With Marine Microalgae *Aurantiochytrium* sp. Rich in n-3 Polyunsaturated Fatty Acids, Frontiers in Marine Science. (2021) 7, 10.3389/fmars.2020.609837, 609837.

[72] Trevi S. , Uren Webster T. M. , Consuegra S. , and García de Leaniz C. , Effects of Micro-Algae Dietary Oil Replacement on Growth, Omega 3 Deposition and Gut Microbiome Composition of Nile Tilapia (*Oreochromis niloticus*), Aquaculture. (2023) 4, no. 3, 10.1002/aff2.164, e164.

[73] Qiao H. , Wang H. , and Song Z. , et al.Effects of Dietary Fish Oil Replacement by Microalgae Raw Materials on Growth Performance, Body Composition and Fatty Acid Profile of Juvenile Olive Flounder, *Paralichthys olivaceus* , Aquaculture Nutrition. (2014) 20, no. 6, 646–653, 10.1111/anu.12127.

[74] Simon A. , Lippemeier S. , and Nowaczek J. , et al.A Fatty Fate: How Different Dietary Microalgae Affect the Fatty Acid Metabolism and Deposition in Rainbow Trout (*Oncorhynchus mykiss*), Aquaculture. (2026) 612, 10.1016/j.aquaculture.2025.743183, 743183.

[75] Szabó Z. , Marosvölgyi T. , and Szabó E. , et al.Effects of Repeated Heating on Fatty Acid Composition of Plant-Based Cooking Oil, Foods. (2022) 11, no. 2, 10.3390/foods11020192. PMC8774349 35053923

[76] Higuera-Ciapara I. , Félix-Valenzuela L. , and Goycoolea F. M. , Astaxanthin: A Review of Its Chemistry and Applications, Critical Reviews in Food Science and Nutrition. (2006) 46, no. 2, 185–196, 10.1080/10408690590957188. 16431409

[77] Lim K. C. , Yusoff F. M. , Karim M. , and Natrah F. M. , Carotenoids Modulate Stress Tolerance and Immune Responses in Aquatic Animals, Reviews in Aquaculture. (2023) 15, no. 2, 872–894, 10.1111/raq.12767.

[78] Pan C. and Chien Y. , Effects of Dietary Supplementation of Alga *Haematococcus pluvialis* (Flotow), Synthetic Astaxanthin and β-Carotene on Survival, Growth, and Pigment Distribution of Red Devil, *Cichlasoma citrinellum* (Günther), Aquaculture Research. (2009) 40, no. 8, 871–879, 10.1111/j.1365-2109.2008.02153.x.

[79] Teimouri M. , Amirkolaie A. K. , and Yeganeh S. , The Effects of *Spirulina platensis* Meal as a Feed Supplement on Growth Performance and Pigmentation of Rainbow Trout (*Oncorhynchus mykiss*), Aquaculture. (2013) 396, 14–19, 10.1016/j.aquaculture.2013.02.009.

[80] Mueller J. , Pauly M. , and Molkentin J. , et al.Microalgae as Functional Feed for Atlantic Salmon: Effects on Growth, Health, Immunity, Muscle Fatty Acid and Pigment Deposition, Frontiers in Marine Science. (2023) 10, 10.3389/fmars.2023.1273614, 1273614.

[81] Ahmed I. , Reshi Q. M. , and Fazio F. , The Influence of the Endogenous and Exogenous Factors on Hematological Parameters in Different Fish Species: A Review, Aquaculture International. (2020) 28, no. 3, 869–899, 10.1007/s10499-019-00501-3.

[82] Seibel H. , Baßmann B. , and Rebl A. , Blood Will Tell: What Hematological Analyses Can Reveal About Fish Welfare, Frontiers in Veterinary Science. (2021) 8, 10.3389/fvets.2021.616955, 616955. 33860003 PMC8042153

[83] Carvalho M. , Marotta B. , and Xu H. , et al.Complete Replacement of Fish Oil by Three Microalgal Products Rich in n-3 Long-Chain Polyunsaturated Fatty Acids in Early Weaning Microdiets for Gilthead Sea Bream (*Sparus aurata*), Aquaculture. (2022) 558, 10.1016/j.aquaculture.2022.738354, 738354.

[84] Le H. , Shao X. , and Krogdahl Å. , et al.Intestinal Function of the Stomachless Fish, Ballan Wrasse (*Labrus bergylta*), Frontiers in Marine Science. (2019) 6, 10.3389/fmars.2019.00140.

[85] Azaza M. , Saidi S. , Dhraief M. , and El-Feki A. , Growth Performance, Nutrient Digestibility, Hematological Parameters, and Hepatic Oxidative Stress Response in Juvenile Nile Tilapia, *Oreochromis niloticus*, Fed Carbohydrates of Different Complexities, Animals. (2020) 10, no. 10, 10.3390/ani10101913, 1913. 33086506 PMC7603184

[86] He C. , Li X. , and Jiang G. , et al.Feed Types Affect the Growth, Nutrient Utilization, Digestive Capabilities, and Endocrine Functions of *Megalobrama amblycephala*: A Comparative Study between Pelleted and Extruded Feed, Fish Physiology and Biochemistry. (2022) 48, no. 4, 1025–1038, 10.1007/s10695-022-01085-1. 35802285

[87] Kong Y. , Li M. , and Chu G. , et al.The Positive Effects of Single or Conjoint Administration of Lactic Acid Bacteria on *Channa argus*: Digestive Enzyme Activity, Antioxidant Capacity, Intestinal Microbiota and Morphology, Aquaculture. (2021) 531, 10.1016/j.aquaculture.2020.735852, 735852.

[88] Chen W. , Gao S. , Chang K. , Zhang Z. , Zhao N. , and Huang Y. , Partial Fishmeal Replacement by Soybean Meal Induces Fish Growth Retardation and Gut Inflammation via Gut Mucosal Barrier Dysfunction and Dysbiosis in Largemouth Bass, Animal Feed Science and Technology. (2024) 316, 10.1016/j.anifeedsci.2024.116067, 116067.

[89] Mota C. S. , Pinto O. , and Sá T. , et al.A Commercial Blend of Macroalgae and Microalgae Promotes Digestibility, Growth Performance, and Muscle Nutritional Value of European Seabass (*Dicentrarchus labrax* L.) Juveniles, Frontiers in Nutrition. (2023) 10, 10.3389/fnut.2023.1165343, 1165343. 37139456 PMC10150028

[90] Galafat A. , Sáez M. I. , and Rodríguez C. , et al.In Vivo valuation of Algae and Their Effect as Dietary Ingredient on Growth, Chemical Composition and Intestinal Functionality in Juvenile Turbot (*Scophthalmus maximus*), Aquaculture. (2024) 592, 10.1016/j.aquaculture.2024.741208, 741208.

[91] Goff L. , Ferrec E. , and Mayer C. , et al.Microalgal Carotenoids and Phytosterols Regulate Biochemical Mechanisms Involved in Human Health and Disease Prevention, Biochimie. (2019) 167, 106–118, 10.1016/j.biochi.2019.09.012. 31545993

[92] Zhu S. , Portman M. , and Cleveland B. , et al.Replacing Fish Oil and Astaxanthin by Microalgal Sources Produced Different Metabolic Responses in Juvenile Rainbow Trout Fed Two Types of Practical Diets, Journal of Animal Science. (2020) 99, no. 1, 10.1093/jas/skaa403, skaa403. PMC8355477 33515472

[93] García-Márquez J. , Galafat A. , and Molina-Roque L. , et al.Microalgal and Cyanobacterial Biomasses Modified the Activity of Extracellular Products From *Bacillus pumilus*: An in Vitro and in Vivo Assessment, Probiotics and Antimicrobial Proteins. (2024) 17, no. 4, 2179–2196, 10.1007/s12602-024-10350-z. 39259377 PMC12405380

[94] Wiśniewski J. , Friedrich A. , Keller T. , Mann M. , and Koepsell H. , The Impact of High-Fat Diet on Metabolism and Immune Defense in Small Intestine Mucosa, Journal of Proteome Research. (2015) 14, no. 1, 353–365, 10.1021/pr500833v. 25285821

[95] Beaumont M. , Portune K. , and Steuer N. , et al.Quantity and Source of Dietary Protein Influence Metabolite Production by Gut Microbiota and Rectal Mucosa Gene Expression: A Randomized, Parallel, Double-Blind Trial in Overweight Humans, American Journal of Clinical Nutrition. (2017) 106, no. 4, 1005–1019, 10.3945/ajcn.117.158816. 28903954

[96] Adeshina I. , Abubakar M. , and Ajala B. , Dietary Supplementation With *Lactobacillus acidophilus* Enhanced the Growth, Gut Morphometry, Antioxidant Capacity, and the Immune Response in Juveniles of the Common Carp, *Cyprinus carpio* , Fish Physiology and Biochemistry. (2020) 46, no. 4, 1375–1385, 10.1007/s10695-020-00796-7. 32232615

[97] Li W. , Huang X. , and Lu X. , et al.Effects of Dietary *Lactobacillus reuteri* on Growth Performance, Nutrient Retention, Gut Health and Microbiota of the Nile Tilapia (*Oreochromis niloticus*), Aquaculture Reports. (2022) 26, 10.1016/j.aqrep.2022.101275, 101275.

[98] Yuan Y. , Omar A. , Emam W. , and Mohamed R. , Impact of Dietary Inclusion of Bile Acid and Fat Percent on Growth, Intestinal Histomorphology, Immune-Physiological and Transcriptomic Responses of Nile Tilapia (*Oreochromis niloticus*), Open Veterinary Journal. (2025) 15, no. 1, 222–243, 10.5455/OVJ.2025.v15.i1.21. 40092181 PMC11910266

[99] Woolley L. , Chaklader M. , and Pilmer L. , et al.Gas to Protein: Microbial Single Cell Protein is an Alternative to Fishmeal in Aquaculture, The Science of the Total Environment. (2022) 859, no. 1, 10.1016/j.scitotenv.2022.160141, 160141. 36395832

