# Supplementary material for: Evaluation of Biorefinery Products From Microchloropsis gaditana, Isochrysis galbana, and Arthrospira platensis in Juvenile Gilthead Seabream (Sparus aurata): Effect on Growth, Nutrient Utilization, Chemical Composition, Metabolism, and Intestinal Functionality
Source: Aquac Nutr. 2026 Aug 6;2026:1957808. doi: 10.1155/anu/1957808 (PMC13447965; doi:10.1155/anu/1957808)
Supplement: Supplementary file 1 [file ANU-2026-1957808-s001.docx]

**Supplementary Figure 1**. Details on microphotographs of the anterior intestine (A) and posterior intestine (B) in juvenile gilthead seabream fed the experimental diets (H&E staining). For each treatment, images taken at different magnifications (from left to right; 4x, 10x, and 40x) are shown and in each case the scale bar is shown at the bottom. CT: control diet; MO50: diet with 50% microalgal oil; MO75: diet with 75% microalgal oil; MO100: diet with 100% microalgal oil.
