# Supplementary material for: Evaluation of Biorefinery Products From Microchloropsis gaditana, Isochrysis galbana, and Arthrospira platensis in Juvenile Gilthead Seabream (Sparus aurata): Effect on Growth, Nutrient Utilization, Chemical Composition, Metabolism, and Intestinal Functionality
Source: Aquac Nutr. 2026 Aug 6;2026:1957808. doi: 10.1155/anu/1957808 (PMC13447965; doi:10.1155/anu/1957808)
Supplement: Supplementary file 2 [file ANU-2026-1957808-s002.docx]

**Supplementary Table 1.** Linear regression analysis (R² and *P*-values) of muscle fatty acid profiles in gilthead seabream fed experimental diets.

| **Fatty acid** | $\boldsymbol{R}^{\boldsymbol{2}}$ | $\boldsymbol{P}_{\boldsymbol{linear regression}}$ |
| --- | --- | --- |
| 14:0 | *0.6634* | *0.6576* |
| 16:0 | *0.2645* | *0.0026* |
| 18:0 | *0.2507* | *0.0041* |
| 16:1n-7 | *0.1110* | *0.0624* |
| 18:1n-9 | *0.2620* | *0.0027* |
| 18:1n-7 | *0.0771* | *0.1240* |
| 18:2n-6 | *0.0787* | *0.1199* |
| 20:4n-6, ARA | *0.0384* | *0.2910* |
| 18:3n-3 | *0.6879* | *0.6573* |
| 18:4n-3 | *0.4020* | *0.1382* |
| 20:4n-3 | *0.1125* | *0.0606* |
| 20:5n-3, EPA | *0.9510* | *< 0.0001* |
| 22:5n-3 | *0.0434* | *0.3070* |
| 22:6n-3, DHA | *0.0049* | *0.7075* |
| ΣSFA | *0.4705* | *< 0.0001* |
| ΣMUFA | *0.2325* | *0.0060* |
| n-3 | *0.1092* | *0.0745* |
| n-6 | *0.4823* | *< 0.0001* |
| n-3/n-6 | *0.9227* | *0.1525* |
| EPA/DHA | *0.9845* | *< 0.0001* |
